# Organizational Citizenship Behavior Predicts Quality, Creativity, and Efficiency Performance: The Roles of Occupational and Collective Efficacies

**DOI:** 10.3389/fpsyg.2020.00758

**Published:** 2020-04-24

**Authors:** Erez Yaakobi, Jacob Weisberg

**Affiliations:** ^1^Business Administration, Ono Academic College, Kiryat Ono, Israel; ^2^Business Administration, Bar-Ilan University, Ramat Gan, Israel

**Keywords:** performance, organizational citizenship behavior, prosocial behavior, collective efficacy, occupational efficacy

## Abstract

Although numerous studies have shown that prosocial behavior impacts performance within organizations, the mechanisms that encourage or discourage these effects have rarely been explored. Two studies were conducted to shed light on the role of psychological beliefs on prosocial dynamics in predicting organizational performance. In Study 1, employees’ beliefs in their inner job-related resources (Occupational Efficacy – OE) were examined as a predictor of OCB. It was posited that OE, which is an inner resource, should positively predict OCB. Study 2 examined whether Collective Efficacy (CE), which is an external resource over which employees have less control, would moderate the OCB-performance prediction. Overall, performance and three core dimensions of performance (quality, creativity and efficiency) were assessed to better capture the specific influence of OCB effects on performance. In Study 1, employees completed inventories measuring their OE, OCB and performance. In Study 2, employees completed inventories measuring their CE and OCB. In addition, their managers completed inventories measuring the CE of their employees’ teams and their employees’ performance. The results of Study 1 revealed that OE emerged as an antecedent of OCB in predicting performance. In Study 2, OCB positively predicted employee performance above and beyond and the effects of their managers’ tenure in position, and CEs. In addition, both employees’ and managers’ CEs moderated the effects of OCB on performance: the performance effects of OCB increased as employees’ and managers’ CE increased, and specifically performance efficiency and performance creativity. These findings contribute to a better theoretical and practical understanding of the core factors that affect the organizational dynamics of prosocial behaviors that can lead to higher performance, and the ways in which OCB positively predicts performance in organizational settings.

## Introduction

Giving and receiving help constitute an integral part of organizational life (Lee et al., 2019). Research on the implications of prosocial behavior in organizations dates back to the 1980s, and has identified three main facets of prosocial behavior: prosocial motives (the willingness to benefit or make an effort for others), prosocial behaviors (gestures that contribute to the welfare of individuals, groups, or organizations), and prosocial impact (attempts to positively influence the lives of others as a result of one’s work).

Although prosocial motives have been discussed in the literature (Bolino and Grant, 2016), few if any empirical studies have explored the role of employees’ beliefs in their inner job-related resources as an antecedent of prosocial behavior. In addition, to the best of our knowledge, no study has examined the moderating effects of employees’ and managers’ beliefs in their work teams on OCB effects on performance.

Motowidlo et al. (1997) argued that knowledge and contextual skills are predictors of OCB. Individuals with high self-efficacy make greater use of adaptive behavioral strategies (Maddux and Lewis, 1995; Raghuram et al., 2003). They are likely to know which citizenship behaviors are appropriate in a workplace situation and how to plan for and deploy these behaviors effectively (Beauregard, 2012). Thus, employees who have high beliefs in their inner resources (i.e., high occupational efficacy-OE) should be more likely to attend voluntary meetings or volunteer to help co-workers with work-related problems because they are better able to proactively plan for these activities and organize their workday to accommodate them. Beauregard (2012) showed that general self-efficacy predicted greater participation in citizenship behaviors in men. Speier and Frese (1997). Morrison and Phelps (1999) found that generalized self-efficacy predicted personal initiative and “taking charge” behavior.

The two studies presented here were designed to explore the role of psychological beliefs on OCBs in predicting organizational performance. Specifically we examined whether OE, which is related to a person’s occupation, would predict employee OCBs better than the general self-efficacy because it is more closely related to the working context and is likely to better capture the psychological beliefs linked to the organizational setting. Study 1 was thus designed to test the hypothesis that OE would predict OCBs. Since the work team acts as one of the main, and frequently the sole, sources of support and assistance to employees in organizations, we posited that the positive effects of prosocial behaviors on performance would be moderated by employees’ and managers’ collective efficacy (CE), a recent extension of Bandura (1997) well-established Efficacy Theory. CE is defined as an “individual’s belief in the capacity of her or his team, department, division, or other relevant organizational unit to execute the courses of action required for performing its mission effectively” (Eden, 2001, p. 79–80). Here we posited that low CE would undermine the performance effects of prosocial behavior, in that work teams whose team members and superiors have low beliefs in their efficacy may become frustrated and thus benefit less from prosocial resources. Study 2 examined how employees’ and managers’ beliefs in their external human resources (CE) moderate the OCB prediction of performance. We conjectured that the influence of OCB on performance would be more closely related to external resources because performance (at least in organizational settings) is becoming increasingly more dependent on group work, synergy and collaboration. Specifically, we hypothesized that higher employee and manager CE would lead to the greater impact of OCB on performance and vice-versa.

## Theoretical Background and Hypothesis Development

### Performance

Companies are cognizant of the financial benefits and competitive edge associated with enhanced employee performance. This involves the ongoing development of high quality innovative goods and services that are delivered on schedule and undercut the competition in terms of price (Miron et al., 2004). When employees’ abilities are aligned with the resources they need to fulfill their goals, performance is enhanced and contributes to firm performance, as do training and motivational perks. Quality, innovation, and efficiency are considered to be the main components of performance in organizations (Miron et al., 2004). Employees are asked to be innovative, while guaranteeing quality output by adhering to company regulations, and working efficiently to meet the constraints imposed by brief delays and tight budgets.

Work also comprises a significant interpersonal component (Blustein, 2004). One of the factors that should lead to better performance within organizations is related to employees’ ability to interact constructively with others, and specifically, their ability to extend and accept assistance in problem solving. An important subset of interactions among employees that can be expected to be related to employee performance is prosocial behavior.

### Prosocial Behavior (OCB)

Resource control theory states that pro-sociality as well as anti-sociality are basic patterns of resource control in human psychological and social functioning (Hawley, 1999). For example, employees need various types of resources (e.g., informational, material, and social) to carry out their tasks in an organization. Interpersonal relations are a source of access to important resources including goal support, know-how and know-who (Ciarrochi et al., 2019). In this sense, friendships on the job should be seen as a resource that individuals strive to develop and maintain (Ciarrochi et al., 2019). It has been argued that good cooperators work better and last longer on the job than poor cooperators (Wilson et al., 2014). Recently, Ciarrochi et al. (2019) suggested that being prosocial is perhaps the best path to success.

One of the core behaviors associated with prosocial behaviors within organizations is Organizational Citizenship Behavior (OCB). OCB is defined as actions that support the social and psychological environment where task performance unfolds (Bolino and Grant, 2016). Lee and Allen (2002) noted that these behaviors represent employees’ voluntary actions such as helping coworkers and attending non-obligatory events which facilitate organizational flow although they are not essential components of the task at hand. OCB across individuals leads to better organizational performance (Choi, 2009). OCB constitutes actions that are taken with no expectation for recognition or compensation (Koslowsky and Pindek, 2011). OCB has been conceptualized as a two-dimensional construct (Williams and Anderson, 1991) made up of OCBO, which comprises behaviors targeting the organization as a whole, and OCBI, which defines behaviors directed toward coworkers.

A significant part of the variance in production and performance quality, quality, efficiency and effectiveness can be explained by interpersonal helping, and specifically OCB (e.g., Organ, 1988; Organ et al., 2006; Podsakoff et al., 2009). OCBs provide social facilitation and reduce social friction by enabling group members to focus on their task more than on interpersonal relationships (Organ, 1988) or conflicts. OCBs potentially increase individual performance efficiencies (e.g., Smith et al., 1983; Borman and Motowidlo, 1997). OCBs can also enhance individuals’ performance by building coordination skills (e.g., Smith et al., 1983). Podsakoff et al. (2000) suggested that OCB can contribute to organizational performance by enhancing coworkers’ and managers’ productivity by facilitating collaboration between work groups and enabling the organization to adapt to environmental changes. Lam et al. (2016) found that engaging in OCB behaviors enhances employees’ vitality, which contributes to the enhancement of employees’ resources leading to better well-being. Based on the above we hypothesized the following:

H1: Employees’ Organizational Citizenship Behavior will positively predict their performance, when measured as: (a) overall performance, (b) performance quality, (c) performance creativity and (d) performance efficiency.

### Efficacy

Employees’ beliefs as to the extent of their control over their work setting can mitigate the negative impact of work demands and have a positive impact on engagement and job performance (Bakker and Demeoruti, 2017; Molero et al., 2018). Employees’ beliefs may also be a useful factor for understanding people’s ability to accept help from others. Self-efficacy, or one’s belief in one’s capacity to execute behaviors required for specific performance using one’s inner resources and the resources in the environment, has been widely studied in Organizational Psychology (Ventura et al., 2015; Barbaranelli et al., 2018). Self-efficacy influences how people behave and how one thinks and feels about the future (Bandura, 1977, 1997). Hence, employees’ self-efficacy beliefs are key to the ways in which they perceive their work context, especially when they face demanding and potentially stressful job demands (Ventura et al., 2015; Molero et al., 2018). Jaeckel et al. (2012) distinguished between two forms of self-efficacy. Generalized self-efficacy is defined as the individual’s belief that s/he can deal effectively with a wide spectrum of everyday problems, a measurable trait that can predict behavior across domains (Chen et al., 2001; Scholz et al., 2002). Task-specific self-efficacy only applies to specific tasks or situations. OE (occupational efficacy) is a specific type of task self-efficacy that characterizes individuals’ confidence in their ability to carry out their duties with success (e.g., Rigotti et al., 2008). OE thus intersects with features of job satisfaction and affective commitment (Schyns and von Collani, 2002). OE is a robust predictor of job performance (Stajkovic and Luthans, 1998; Rigotti et al., 2008).

Employees’ generalized self-efficacy was reported to exhibit a positive association with OCB (Jawahar et al., 2008). OE is likely to be associated with OCB since it is part of the self-regulation designed to control one’s own behavior and expend more effort (Bandura, 1977). OCBs are strengthened by impression management, where people try to present a good image of themselves to others, and prosocial motives (Grant and Mayer, 2009) that require self-regulatory efforts (Vohs et al., 2005). OE also enhances personal initiative, an important facet of OCB (Speier and Frese, 1997), such that workers with high initiative contribute to their organization’s aims and long-term goals by engaging in more proactive actions (Frese et al., 1997). Hence, we expected that OE, a type of self-efficacy belief that is strongly related to the work context, would be a core antecedent and predictor of OCBs. This hypothesis was tested in Study 1.

H2: Employees’ Occupational Efficacy will positively predict their Organizational Citizenship Behaviors.

In today’s highly interdependent work arena, employees’ behaviors are affected by other team members. We thus hypothesized that OCB effects on employees’ performance would be moderated by employees’ and their managers’ beliefs regarding the work team to which the employees belong; namely, their collective efficacy.

### Forms of Collective Efficacy

Eden argued that “self-efficacy is only half of the efficacy story” (Eden et al., 2010, p. 688). Self-efficacy beliefs are complemented by external efficacy, which is a person’s beliefs about available human or inanimate resources that help or undermine performance (Stirin et al., 2012). These range from equipment, tools, effective guidance and support to good working conditions, a superior starting point, and other facilitators (Eden, 2001). External efficacy covers the capacities of one’s group, the availability of means, and the circumstances at hand. The belief that external sources will provide assistance that results in better performance depends to a large extent on one’s belief in the abilities of others from whom such assistance and support are received. Because OCB is a social phenomenon, collective efficacy should moderate OCB effects on performance.

CE is a specific type of external efficacy and was first defined as “the shared perception of a group of its efficacy to perform a behavior and to organize and execute the actions required to reach certain levels of achievement” (Bandura, 1997, p. 447; Martínez et al., 2011). Eden (2001) defined CE as an “individual’s belief in the capacity of her or his team, department, division, or other relevant organizational unit to execute the courses of action required for performing its mission effectively.” A meta-analysis reported a strong positive association between CE and group performance (Stajkovic et al., 2009). Team efficacy mediated the association between the ability-enhancing practices of one’s team and team creativity (Ma et al., 2017).

CE is considered a key predictor of performance in a variety of collective settings, including work teams (Salanova et al., 2014). CE significantly predicted less error variance in nursing tasks (Lee and Ko, 2010). Studies have also shown that CE is positively associated with self-reported innovations (Salanova et al., 2012) as well as people’s actual innovativeness (Choi and Chang, 2009) and decision-making quality (Lam and Schaubroeck, 2011).

The antecedents of CE may be a function of enactive mastery, where confidence accumulates gradually as teams get feedback on their specific job performance, as well as through vicarious experience and verbal persuasion (Gist and Mitchell, 1992; Tasa et al., 2007). Gist and Mitchell (1992) suggested that there is a recursive relationship between past performance and CE since positive feedback on challenging tasks can result in stronger efficacy beliefs, which then can promote greater success. Tasa et al. (2007) reasoned that CE develops in part via exchanges of information and observed behaviors within a team, and that CE is affected by the total amount of teamwork behaviors in a team. Other findings suggest that a team manager may also affect CE beliefs. Goncalo et al. (2010) showed that when there is high group confidence in the initial phase of a group experience, there is less likelihood of process conflict, which nevertheless can be advantageous in the first phases of a project. The group manager becomes a key resource for feedback and evaluations of the group which impacts their CE as well. Recently, Pak and Kim (2018) emphasized the importance of team managers’ role in facilitating performance. They further pointed to the team manager as a primary interpretive filter who enables team members to identify differences in high performance work system intensity, which in turn affects team performance. Therefore, we suggest the following hypotheses:

H3: Employees’ CE will positively predict their (a) overall performance, (b) performance quality, (c) performance creativity, and (d) performance efficiency.
H4: Managers’ CE will positively predict employees’ (a) overall performance, (b) performance quality, (c) performance creativity, and (d) performance efficiency.

Scholars have provided some possible reasons for the positive associations between employee OCBs and performance evaluations. Studies have shown that employees’ OCBs are interpreted by managers as behavioral manifestations of loyalty and/or loyalty commitment (Allen and Rush, 1998), although managers may tend to simply like these individuals more (Lefkowitz, 2000). Employees who exhibit OCBs may receive higher evaluations by managers as a form of reciprocity (Podsakoff et al., 1993). It has also been argued that OCBs are positively related to other individuals such as through reward allocations and to the unit-level such as the quality and quantity of product outcomes and profitability, and can explain a significant fraction of the variance in job performance ratings compared to employees’ task performance ratings (Podsakoff et al., 2009). Therefore, we suggest the following hypotheses:

H5: Employees’ Organizational Citizenship Behavior will only positively predict their overall performance, performance quality, performance creativity, and performance efficiency when they have high collective efficacy but not when they have low collective efficacy.
H6: Employees’ Organizational Citizenship Behavior will only positively predict their overall performance, performance quality, performance creativity, and performance efficiency when their manager’s collective efficacy is high but not when their manager’s collective efficacy is low.
H7: Employees’ Organizational Citizenship Behavior will predict performance more strongly when both employees’ and managers’ collective efficacy beliefs are high than when employees’ collective efficacy beliefs are high and managers’ collective efficacy beliefs are low or vice versa. Employees’ Organizational Citizenship Behavior will not predict performance when both managers’ and employees’ CE are low.

## The Current Studies

**FIGURE 1:**
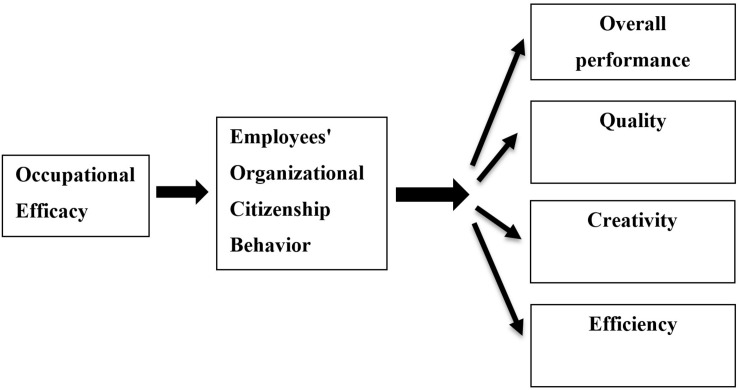
Mediation effect of OCB on the association between occupational efficacy and performance (Study 1).

Study 1 examined whether employees’ OCB would predict overall performance, performance quality, performance creativity, and performance efficiency. In this study, we also examined the antecedents of OCB in terms of OE. We focused on intra-individual processes by hypothesizing that OE, which relates to an internal quality, would best predict OCBs. Figure 1 presents the research model for Study 1.

**FIGURE 2:**
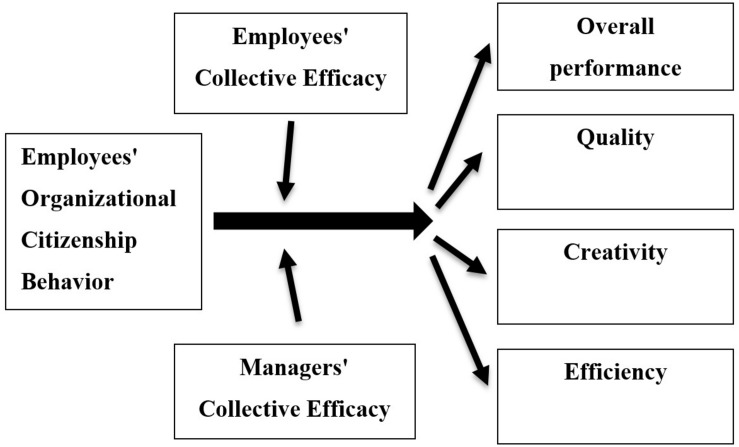
Moderating effect of employees’ and managers’ collective efficacy on the association between employees’ OCB and facets of performance (Study 2).

Study 2 consisted of an empirical examination of the factors that strengthen or inhibit the effects of OCB on the four performance measures above. Specifically, we examined whether employees’ and managers’ CE would moderate the OCB effects on the four performance measures. In addition, to control for common method bias, in Study 2 we collected data from employees and their managers, where managers evaluated their employees’ performance. We hypothesized that OCB, which is a social phenomenon, would predict performance moderated by more social external processes, and specifically the CE of employees and managers. Dyads of managers-employees were also considered in conceptualizing how CE impacts performance. Figure 2 presents the research model for Study 2.

All participants were salaried employees who volunteered to take part in this study. All worked full time and were enrolled in a weekend M.B.A. program at one of two leading academic institutions in Israel.

## Study 1

Study 1 examined stage one of the model. In this study we examined whether OCB would predict all three dimensions of employee performance (quality, creativity, efficiency) above and beyond demographic effects, and explored one of the core antecedents of prosocial behaviors in predicting performance. Specifically, we examined whether OE serves as an antecedent to prosocial behavior (OCB) using a mediation model of OCB on OE effects on performance. These were hypothesized since previous findings have revealed that OCB positively predicts performance (e.g., Lam et al., 2016; Park, 2018; Germeys et al., 2019). Moreover, job self-efficacy was found to serve as a core antecedent of OCB in customer service employees (Reizer and Hetsroni, 2015). Here we combined these two findings and examined both concurrently and in terms of the three core performance facets rather than only one general measure.

### Method

#### Sampling and Subjects

One hundred twenty two employees agreed to participate. All worked full time. Of the employees, 44% were men, with ages ranging from 22 to 62 (mean age = 37.59). Experience in their profession ranged from 1 to 26 years (mean = 6.19), tenure in their position ranged from 1 to 38 years (mean = 11.3) and tenure in their current organization ranged from 1 to 47 years (mean = 12.89). Most of the participants worked in high-tech industries as engineers. All participants signed an informed consent form before filling in the questionnaires and were instructed that they could withdraw at any time without penalty. No one did so.

Most of the employees were tenured, had enough experience in the labor market, and represented a diverse range of professions. All participants completed questionnaires during class at a major Israeli academic institution in 2018–2019 for a response rate of 100%.

#### Materials and Procedure

##### Performance

Performance was assessed on the well-established Miron et al. (2004) Inventory, which comprises 14 items tapping employee quality, creativity and efficiency on a 7-point Likert-type scale ranging from 1 (*strongly disagree*) to 7 (*strongly agree*). The questionnaire consists of three subscales assessing *quality* [e.g., Thorough in work, Adheres to rules, Does not cut corners (α = 0.83)], *creativity* [e.g., Finds unusual solutions, Implements new ideas (α = 0.89)] and *efficiency* [e.g., Attends to matters of efficiency and saving, Keeps to planned schedule (α = 0.82)]. The measure of performance was found to be valid (Miron et al., 2004; Yaakobi and Weisberg, 2018).

##### Organizational citizenship behavior

OCB was defined here in terms of the target or beneficiary of citizenship behavior. The Smith et al. (1983) altruism and compliance subscales have been used elsewhere to assess OCBI and OCBO (I = directed toward other individuals in the workplace; O = directed toward the organization; Farh et al. (1990), and Williams and Anderson (1991) also differentiated between OCBI and OCBO. Since we focused on individual CE beliefs, we only administered the OCBI measure. Eight items reflecting OCBI were used (see Appendix). Employees indicated how frequently they engaged in these behaviors on a 7-point scale ranging from 1 (*never*) to 7 (*always*). Confirmatory factor analysis clearly showed that the OCBI-factor model was a one-factor model, which thus lends weight to a single OCBI measure (α = 0.83).

##### Occupational efficacy

The OE questionnaire (Horovitz, 2012) was used to measure employees’ OE (job-specific efficacy at the individual level). Employees evaluated their efficacy at work. Participants ranked OE on a 5-point Likert-type scale that ranged from 1 (*strongly disagree*) to 5 (*strongly agree*). Sample items were “I can persuade any employer to hire me”; “I can learn new demands at work quickly” (α = 0.76).

##### Control variable

Employees with longer job tenure have more work experience and may perform better. The same logic may apply to tenure in the organization and tenure in the profession. In addition, employees’ gender and age were examined in the analyses to capture the main demographic variables. The study was approved by the institutional review board.

### Results

A correlational analysis revealed that employees’ organizational citizenship behaviors were positively associated with their performance (see Table 1).

**TABLE 1:** Means, standard deviations, and inter-correlations between variables (Study 1).

	Mean	*SD*	1	2	3	4	5	6	7	8	9
1. OCB	4.10	0.68	–								
2. Overall performance	5.54	0.94	0.28**	–							
3. Performance–quality	5.70	0.99	0.08	0.77***	–						
4. Performance–creativity	5.26	1.21	0.19*	0.86***	0.50***	–					
5. Performance–efficiency	5.62	1.19	0.36***	0.80***	0.53***	0.60***	–				
6. Age	37.59	8.57	0.18	0.18	0.10	0.14	0.11	–			
7. Tenure in organization	12.89	10.21	0.24*	0.12	0.01	0.10	0.20*	0.83***	–		
8. Tenure in position	6.19	5.97	0.24*	0.05	0.02	0.03	0.11	0.58***	0.64***	–	
9. Tenure in profession	11.30	7.93	0.12	0.16	0.13	0.13	0.04	0.73***	0.62***	0.62***	–
10. Gender	0.43	0.50	–0.08	0.14	0.04	0.19*	0.01	0.07	0.02	–0.02	0.13

Gender: 1 = male, 0 = female; Tenure appears in years *p < 0.05, **p < 0.01, ***p < 0.001.

When examining the three performance dimensions, the correlational analyses revealed that OCB was positively associated with both performance creativity and efficiency but not with performance quality. Regarding demographics, only tenure in the organization and gender were positively correlated to performance, but only to specific dimensions (Table 1).

A regression analysis was performed to better capture the predictive power of OCB on performance dimensions above and beyond employees’ demographic effects. In the first step of the regression analysis, employees’ tenure in their organization, tenure in their profession, and gender were entered simultaneously into the equation. The decision to enter these demographics was based on the results of the correlational analyses. Kidder (2002) also found that women engage in more OCBI than men.

In step two, employees’ OCB was entered into the equation (see Table 2).

**TABLE 2:** Hierarchical regression analysis predicting performance by OCB (Study 1).

Performance	Overall performance		Quality		Creativity		Efficiency	
Effects	Step 1	Step 2	Step 1	Step 2	Step 1	Step 2	Step 1	Step 2
Tenure in organization	0.03	–0.06	–0.13	–0.14	–0.02	–0.09	0.32*	0.23
Tenure in profession	0.17	0.19	0.27*	0.27*	0.17	0.18	–0.12	–0.10
Gender	0.14	0.17	0.003	0.01	0.19	0.21*	0.04	0.07
Δ*R*^2^	0.07		0.05		0.07		0.07	
OCB		0.27**		0.04		0.23*		0.29**
Δ*R*^2^		0.07*		0.001		0.05*		0.08**
Total *R*^2^		0.13***		0.05		0.12*		0.15**

Gender: 1 = male, 0 = female *p < 0.05, **p < 0.01, ***p < 0.001.

As can be seen in Table 2, none of the demographic variables were associated with employees’ overall performance. However, OCB was strongly associated with employees’ overall performance, above and beyond demographic effects, thus supporting H1. With respect to the three dimensions of performance, OCB positively predicted performance creativity and performance efficiency above and beyond demographic effects, but in line with the results of the correlational analysis, OCB did not predict performance quality.

To examine whether OE was a prerequisite for OCB concurrently with the prediction of OCB on performance we used the PROCESS macro (Hayes, 2013) to calculate two sets of regressions^1^. The first set tested for an association between occupational efficacy and OCB. The second set examined the relationship of OCB and performance. To test the significance of the indirect effects of OE on performance via OCB, the bootstrapping approach was used. The 95% *CI* for the indirect effects was calculated on 5,000 resamples (Hayes, 2013). We conducted 4 sets of analyses, one for the overall performance measures, and three for the three performance dimensions (quality, creativity and efficiency).

Table 3 presents the results. With respect to the overall performance measure, OE was positively associated with OCB, as indicated by the significant unstandardized regression coefficient. Supporting our hypotheses, there was a positive association between OCB and performance, when controlling for the OE effects. OE had an indirect effect on performance. The two-tailed significance test (assuming a normal distribution) indicated that there was an indirect effect of OE on performance. The bootstrapped 95% *CI* around the indirect effect did not include zero.

**TABLE 3:** Regression result for simple mediation (Study 1).

Variable	β	*SE*	*t*	*p*
**Overall performance**				
**Direct and total effects**				
P regressed on OE	0.04	0.09	0.44	0.0662
OCB regressed on OE	0.21	0.09	2.36	0.020
P regressed on OCB, controlling for OE	0.30	0.10	3.07	0.003
	**β**	***SE***	***LLCI*95%**	***ULCI*95%**
**Indirect effects and significance using normal distribution**				
**Bootstrap results for indirect effects**				
Effect	0.063	0.022	0.012	0.163
**Performance quality**				
**Direct and total effects**				
P regressed on OE	0.08	0.10	0.85	0.399
OCB regressed on OE	0.21	0.09	2.36	0.020
P regressed on OCB, controlling for OE	0.09	0.10	0.90	0.370
P regressed on OE controlling for OCB	0.08	0.10	0.85	0.400
	**β**	***SE***	***LLCI*95%**	***ULCI*95%**
**Indirect effects and significance using normal distribution**				
**Bootstrap results for indirect effects**				
Effect	0.02	0.03	–0.024	0.084
**Performance creativity**				
**Direct and total effects**				
P regressed on OE	0.08	0.09	0.92	0.362
OCB regressed on OE	0.21	0.09	2.36	0.020
P regressed on OCB, controlling for OE	0.24	0.10	2.45	0.016
	**β**	***SE***	***LLCI*95%**	***ULCI*95%**
**Indirect effects and significance using normal distribution**				
**Bootstrap results for indirect effects**				
Effect	0.05	0.04	0.003	0.152
**Performance efficiency**				
**Direct and total effects**				
P regressed on OE	0.06	0.09	0.65	0.518
OCB regressed on OE	0.25	0.09	3.72	0.0003
P regressed on OCB, controlling for OE	0.32	0.09	2.76	0.007
	**β**	***SE***	***LLCI*95%**	***ULCI*95%**
**Indirect effects and significance using normal distribution**				
**Bootstrap results for indirect effects**				
Effect	0.08	0.05	0.013	0.241

*P, performance; OE, Occupational efficacy; Unstandardized regression coefficients are reported. Bootstrap sample size = 5,000. LL, lower limit; CI, confidence interval; UL, upper limit.*

With respect to the performance quality measure, OE was positively associated with OCB, as indicated by the significant unstandardized regression coefficient. However, in contrast to our hypothesis on performance quality, no significant positive association was found between OCB and performance when controlling for the OE effects. The indirect effect of OE on performance failed to reach significance. The two-tailed significance test (assuming a normal distribution) indicated that there was no indirect effect of OE on performance. The bootstrapped 95% *CI* around the indirect effect included zero.

With respect to the performance creativity measure, OE was positively associated with OCB, as indicated by the significant unstandardized regression coefficient. Consistent with our hypothesis, there was a positive association between OCB and performance when controlling for the OE effects. OE had an indirect effect on performance. The two-tailed significance test (assuming a normal distribution) indicated that the indirect effect was significant. The bootstrapped 95% *CI* around the indirect effect did not include zero.

With respect to the performance efficiency measure, OE was positively associated with OCB, as shown by the significant unstandardized regression coefficient. In support of our hypothesis, there was a positive association between OCB and performance when controlling for OE effects. OE had an indirect effect on performance. The two-tailed significance test (assuming a normal distribution) indicated that the indirect effect was significant. Bootstrap results showed that the bootstrapped 95% *CI* around the indirect effect did not include zero.

### Discussion

Study 1 suggested that OCB predicted the general performance measure and specifically creativity and efficiency performances above and beyond demographic effects but not the quality performance measure. This findings leads to a more accurate and in-depth understanding of the specific effects of OCB on specific performance facets than has been reported in the literature for general performance (Choi, 2009; Lam et al., 2016; Park, 2018; Germeys et al., 2019). In addition, OE appeared to be a core antecedent of prosocial behaviors in predicting performance for the general performance measure and specifically creativity and efficiency performance, but not the quality performance measure. The findings that OE serves as a core antecedent of OCB is consistent with previous findings on job self-efficacy and OCB in customer service employees (Reizer and Hetsroni, 2015).

## Study 2

Study 2 was conducted to examine the next step in the model and specifically, the moderating roles of CE as assessed separately for managers and employees in OCB effects on performance. Our main hypothesis was that the OCB prediction on the three performance facets would be stronger when employees’ and managers’ score high on CE. These hypotheses were based on previous findings that pointed to the important role of beliefs in team efficacy (CE) on performance (Myers et al., 2004; Salanova et al., 2014) coupled with the finding that team managers play a prime role in facilitating performance (Pak and Kim, 2018). Moreover, to control for possible common method bias, in Study 2 employees’ performance was evaluated by their managers.

### Method

#### Participants

Ninety managers and their employees (a total of 180 participants) agreed to participate. All worked full time. Of the managers, 62% percent were men, with ages ranging from 23 to 72 (mean age = 37.62), their tenure in their profession ranged from 0.5 to 30 years (mean = 5.27), their tenure in their position ranged from 0.5 to 25 years (mean = 2.09), and their tenure in the current organization ranged from 0.5 to 22 years (mean = 4.21).

Fifty-two percent of the employees were men, with ages ranging from 19 to 57 (mean = 36.69), their tenure in their profession ranged from 0.5 to 20 years (mean = 4.42), their tenure in their position ranged from 0.5 to 15 years (mean = 2.29), and their tenure in the current organization ranged from 0.5 to 18 years (mean = 2.85). Thus, most of the managers and employees were tenured and had sufficient experience in the labor market. They represented a variety of professions, and most were working in high-tech industries as engineers. All participants completed the surveys in class at a major Israeli academic institution in 2018–2019 (100% response rate).

#### Materials and Procedure

##### Performance

To avoid possible self-report-biases, the managers were asked to evaluate their employees’ performance on the same well-established measure developed by Miron et al. (2004) used in Study 1. The reliability of the performance measures assessed by managers was α = 0.89 for overall performance, α = 0.84 for performance quality, α = 0.93 for performance creativity, and α = 0.82 for performance efficiency.

##### Organizational citizenship behavior

Study 2 used the same measure as in Study 1 (α = 0.92).

##### Collective efficacy

The CE scale (Guzzo et al., 1993) was used by managers and employees to assess CE. This questionnaire is composed of 15 items assessing various beliefs regarding team or group performance. These items are scored on a 5-point Likert-type scale ranging from 1 (*strongly disagree*) to 5 (*strongly agree*). In the original measure, participants assess themselves. In the current study, employees evaluated the efficacy of their respective work teams, and managers evaluated the efficacy of a target employee’s work team. Sample items included “I believe that the employees in my/my employee’s team will have high productivity if they work hard” and “I believe that the employees in my/my employee’s team can solve any problem they face” (α = 0.93).

##### Control variable

Based on studies that found that managers’ tenure potentially affects their employees’ performance (Drazin and Rao, 1999; Goldsmith, 2013; Chen et al., 2017), this study used managers’ tenure in their profession as a control variable. Managers with longer tenure in their profession have more work experience that may have an effect on their employees’ performance.

### Results

We first conducted a correlational analysis between performance, employees’ CE, managers’ evaluations of CE, and managers’ tenure in their profession^2^. Table 4 presents the means, standard deviations, and correlations.

**TABLE 4:** Means, standard deviations, and inter-correlations between variables (Study 2).

	Mean	*SD*	1	2	3	4	5	6	7
1. OCB	4.25	0.83	–						
2. Employees’ CE	5.80	1.24	0.12	–					
3. Managers’ CE	5.77	0.85	0.11	0.59***	–				
4. Tenure in the profession	5.18	6.03	−0.37**	–0.01	–0.001	–			
5. Overall performance	5.29	0.85	0.29**	0.21*	0.43***	0.04	–		
6. Performance-quality	5.76	0.80	0.09	0.10	0.19	0.04	0.63**	–	
7. Performance-creativity	4.80	1.39	0.21*	0.20	0.40**	0.03	0.81***	0.20**	–
8. Performance-efficiency	5.41	1.02	0.24*	0.22*	0.34**	0.07	0.76***	0.47***	0.43***

CE, Collective Efficacy; Tenure appears in years *p < 0.05, **p < 0.01, ***p < 0.001.

As shown in Table 4, tenure was unexpectedly found to be negatively associated with OCB. One possible explanation is that the tenure of the older participants led them to engage in less OCB due to burnout or fatigue, although not empirically controlled for here. With respect to the main hypotheses, employees’ CE was significantly and positively associated with employees’ overall performance and performance efficiency. Managers’ evaluations of CE were positively associated with employees’ overall performance, performance efficiency, and performance creativity. In line with Study 1, the findings of Study 2 supported H1 with respect to the overall performance measure. It also enabled a better, more in-depth understanding of the specific performance dimensions related to OCB. The analyses also supported H3 and H4 regarding the impact of CE on performance for the general performance measure and specifically for the efficiency performance measure and for managers’ CE on the creativity performance measure^3^.

To examine the integrated model, which included the moderating roles of both employees’ CE and their managers’ evaluation of CE in employees’ OCB effects on performance, we used the PROCESS macro (Hayes, 2013) Model 3. We conducted four analyses: one for the overall performance measure and three additional analyses for the three performance dimensions. To test the significance of the effects, and calculate the 95% CI for the indirect effects, bootstrapping with 5,000 resamples (Hayes, 2013) was used (see Table 5).

**TABLE 5:** β, SE, t, p, and 95% confidence interval values for the analysis of overall performance as a function of OCB on ± 1 SD of managers’ and employees’ collective efficacies (Study 2).

Predictor	β	*SE*	*t*	*p*	95%LCI	95%UCI	β	*SE*	*t*	*p*	95%LCI	95%UCI
**Overall performance**							**Performance quality**					
Constant	0.01	0.09	0.09	0.932	–0.17	0.18	–0.12	0.11	1.16	0.249	–0.33	0.09
Managers’ CE (MCE)	0.43	0.11	3.81	0.001	0.21	0.66	0.25	0.13	1.87	0.066	–0.02	0.52
Employees’ CE (ECE)	–0.01	0.13	–0.07	0.946	–0.26	0.24	0.18	0.15	1.20	0.235	–0.12	0.48
OCB	0.18	0.10	1.87	0.065	–0.01	0.37	–0.08	0.11	0.74	0.463	–0.31	0.14
OCB × ECE	0.50	0.15	3.39	0.001	0.21	0.80	0.65	0.18	3.69	0.001	0.30	1.00
OCB × MCE	0.19	0.11	1.70	0.094	–0.03	0.41	0.17	0.13	1.32	0.192	–0.09	0.43
ECE × MCE	–0.05	0.08	–0.70	0.489	–0.21	0.10	0.11	0.09	1.21	0.230	–0.07	0.29
OCB × ECE × MCE	–0.33	0.16	2.01	0.048	–0.65	–0.01	–0.09	0.19	–0.46	0.650	–0.47	0.30
Tenure in position	0.18	0.09	1.97	0.053	–0.01	0.35	0.11	0.11	1.07	0.287	–0.10	0.32
**Performance–creativity**							**Performance–efficiency**					
Constant	0.02	0.10	0.21	0.83	–0.18	0.22	0.01	0.11	0.05	0.958	–0.20	0.21
Managers’ CE (MCE)	0.34	0.13	2.70	0.009	0.09	0.60	0.41	0.13	3.03	0.003	0.14	0.68
Employees’ CE (ECE)	0.02	0.14	0.15	0.884	–0.26	0.30	–0.11	0.15	0.73	0.467	–0.41	0.19
Employees’ OCB (EOCB)	0.12	0.11	1.16	0.250	–0.09	0.34	0.25	0.11	2.21	0.031	0.02	0.48
EOCB × ECE	0.38	0.17	2.30	0.024	0.05	0.71	0.40	0.18	2.28	0.025	0.05	0.75
EOCB × MCE	0.18	0.12	1.44	0.154	–0.07	0.42	0.18	0.13	1.38	0.173	–0.08	0.44
ECE × MCE	–0.05	0.09	–0.57	0.569	–0.22	0.12	–0.19	0.09	2.03	0.064	–0.37	–0.01
EOCB × ECE × MCE	–0.32	0.18	1.75	0.084	–0.68	0.04	–0.48	0.19	2.50	0.015	–0.87	–0.10
Tenure in position	0.12	0.10	1.23	0.22	–0.08	0.32	0.21	0.11	2.03	0.046	0.01	0.43

The findings of Study 2 and the correlational analysis showed that employees’ OCB was positively associated with their performance efficiency, above and beyond the effects of managers’ tenure in their position and employees’ and managers’ CE. Moreover, the significant interaction effects suggested that the association between OCBs and performance was dependent on CE beliefs. Specifically, a significant two-way interaction was found between employees’ OCB and their CE beliefs in predicting overall performance, and a significant three-way interaction was found between employees’ OCB, their CE beliefs, and their managers’ CE beliefs in predicting overall performance. Three significant two-way interactions between employees’ OCB and their CE beliefs were found to predict the three performance dimensions (quality, creativity, and efficiency). Thus, the OCB association to performance emerged as dependent on employees’ beliefs in the capabilities of their team to perform its tasks. Furthermore, a significant three-way interaction between employees’ OCB, employees’ CE and managerial CE emerged for the efficiency performance measure.

To probe the essence of these interactions, simple slope analyses (Aiken and West, 1991) were used for each interaction. In each analysis, the moderator was analyzed at plus/minus one SD from the mean as shown in Table 6.

**TABLE 6:** β, SE, t, p, and 95% confidence interval values for the analysis of performance as a function of OCB on ± 1 SD of managers’ and employees’ collective efficacies (Study 2).

Managers’ CE	Employees’ CE	β	*SE*	*t*	*p*	95%LCI	95%UCI
**Overall Performance**							
−1	−1	–0.79	0.27	2.91	0.005	–1.34	–0.25
−1	0	0.02	0.16	0.12	0.902	–0.30	0.34
−1	1	0.83	0.29	2.84	0.006	0.25	1.42
0	−1	–0.30	0.18	1.69	0.096	–0.66	0.06
0	0	0.19	0.10	2.04	0.045	0.004	0.38
0	1	0.69	0.17	4.01	0.001	0.35	1.03
1	−1	0.19	0.28	0.67	0.502	–0.37	0.75
1	0	0.37	0.12	3.02	0.004	0.13	0.61
1	1	0.55	0.17	3.21	0.002	0.21	0.89
**Performance Efficiency**							
−1	−1	–0.76	0.32	2.35	0.021	–1.40	–0.12
−1	0	0.10	0.19	0.52	0.603	–0.28	0.48
−1	1	0.96	0.35	2.76	0.007	0.27	1.65
0	−1	–0.13	0.21	0.62	0.541	–0.55	0.29
0	0	0.26	0.11	2.33	0.023	0.04	0.49
0	1	0.65	0.20	3.21	0.002	0.25	1.06
1	−1	0.50	0.33	1.51	0.137	–0.16	1.16
1	0	0.42	0.14	2.95	0.004	0.14	0.71
1	1	0.35	0.20	1.75	0.025	–0.05	0.75

#### Probing the interactions for the overall performance measure

**FIGURE 3:**
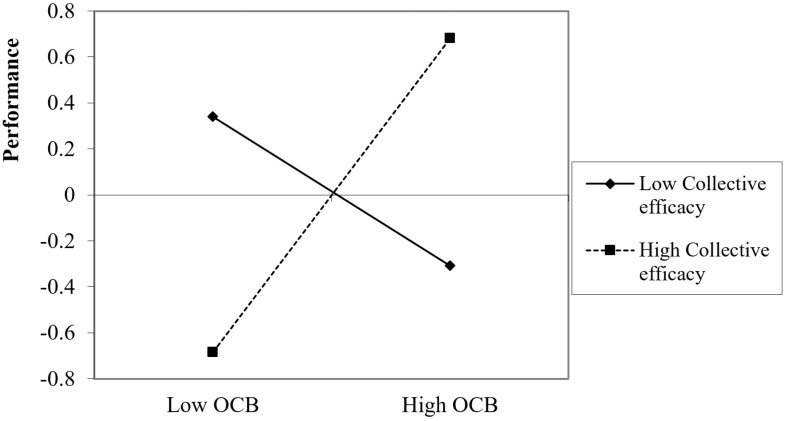
Impact of OCB on overall performance as a function of employees’ collective efficacy.

The employees’ OCB x CE interaction analysis to predict their overall performance indicated that OCB was positively associated with employee performance only when their CE was high (+1*SD*), β = 0.83, *p* < 0.001 [0.50, 1.17] or moderate (*SD*), β = 0.26, *p* = 0.023 [0.04, 0.47] but not when their CE was low (−1*SD*), β = −0.32, *p* = 0.129 [−0.74, 0.09] (Figure 3), thus supporting H5. These findings thus suggest that OCB only positively predicts performance when employees have a moderate or strong belief in their team’s capacity to perform its tasks, but not when they have low beliefs in their team’s abilities. When employees do not believe that their team has high abilities, OCB did not have a significant effect on performance.

**FIGURE 4:**
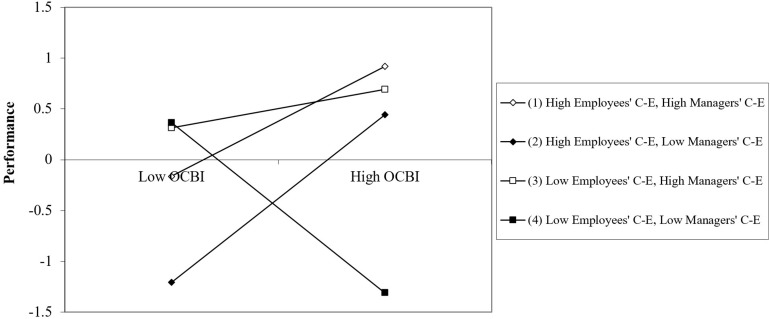
Impact of OCB on overall performance as a function of employees’ collective efficacy (CE).

The results of a three-way interaction probe that separately analyzed the effects of OCB on overall performance for the different CE values (±1 SD of each CE measure) appear in Table 5. Figure 4 provides a graphic representation of the results of the three-way interaction.

As can be seen in Table 6 and Figure 4, OCB positively predicted performance for (a) high employee CE values, regardless of their managers’ CE or (b) moderate employee CE values when their managers’ CE was high or moderate. This was not the case when managers’ CE was low. Moreover, when both employees and managers had low CEs, OBC was associated with negative effects on performance, possibly due to frustration.

#### Probing the interactions for dimensions of performance

**FIGURE 5:**
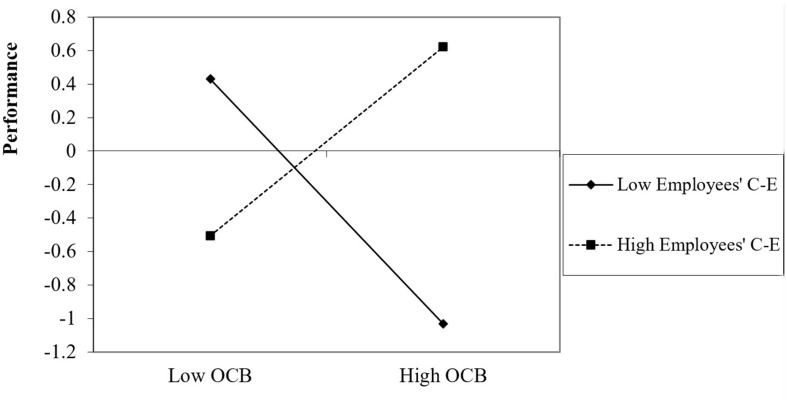
Impact of OCB on performance quality as a function of employees’ collective efficacy.

With respect to performance quality, the analysis revealed that employees who were high on OCB and on CE performed significantly better than participants who were low on OCB, β = 0.75, *p* < 0.001 [0.42, 1.15]. In contrast, employees who were low on CE performed more poorly than when they were high on OCB, β = −0.74, *p* = 0.002 [−1.21, −0.27] than when low on OCB. The results for the moderate level of employees’ CE were not significant, β = 0.12, *p* = 0.966 [−0.24, 0.25] (Figure 5).

Thus, in line with findings for the overall performance measure, high employee CE is important for leveraging OCB to improve performance. Even more dramatically, when employees do not believe in their team’s abilities, OCB leads to lower performance quality.

**FIGURE 6:**
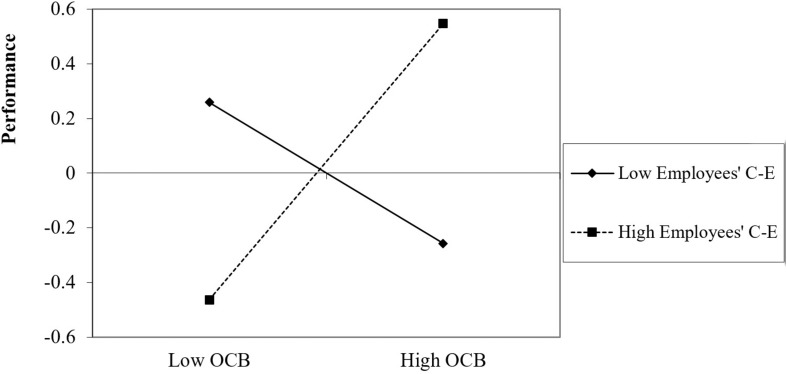
Impact of OCB on performance creativity as a function of employees’ collective efficacy.

With respect to performance creativity, the analysis revealed that employees high on both OCB and CE performed significantly better than employees who were low on CE, β = 0.62, *p* = 0.001 [0.26, 0.97]. Unlike the results for performance quality, among employees with low or moderate CE, the effects of OCB on performance creativity were not significant, β = −0.26, *p* = 0.244 [−0.70, 0.18] and β = 0.18, *p* = 0.131 [−0.05, 0.41], respectively (Figure 6).

Thus, with respect to overall performance and performance quality, employees’ positive beliefs in their team are necessary to enable OCB to predict performance. In contrast to performance quality, low CE does not lead to the opposite results of OCB on performance, but rather to non-significant effects.

**FIGURE 7:**
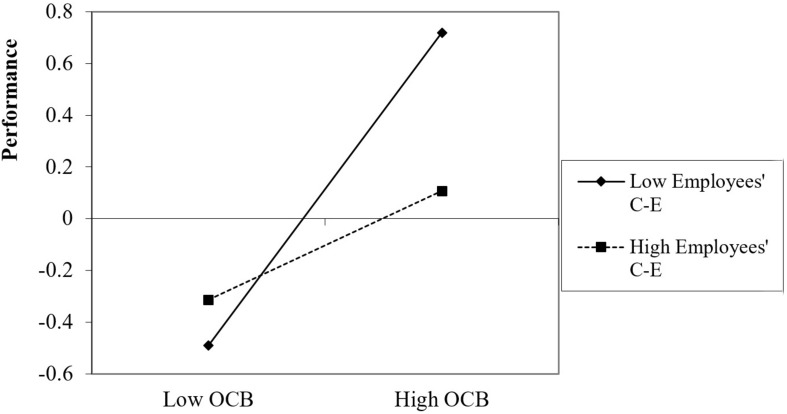
Impact of OCB on performance efficiency as a function of employees’ collective efficacy.

With respect to performance efficiency, the analysis revealed that employees who were high on OCB and on CE performed significantly better than employees who were low on OCB, β = 0.70, *p* = 0.001 [0.31, 1.09]. Even for moderate levels of CE, OCB significantly and positively predicted performance efficiency, β = 0.29, *p* = 0.029 [0.03, 0.53]. For employees who were low on CE, OCB had non-significant effects on creative performance, β = −0.13, *p* = 0.585 [−0.62, 0.35] (Figure 7).

With respect to performance efficiency, employees’ positive or moderate beliefs in their team are needed to enable OCB to predict performance. However, unlike the performance quality facet, low CE did not lead to the opposite results of OCB on performance, but rather to non-significant effects.

Finally, the results for the three-way interaction were probed by separately analyzing the effects of OCB on the efficiency performance measure for different CE values (± 1 SD of each CE measure). The findings appear in Table 6.

**FIGURE 8:**
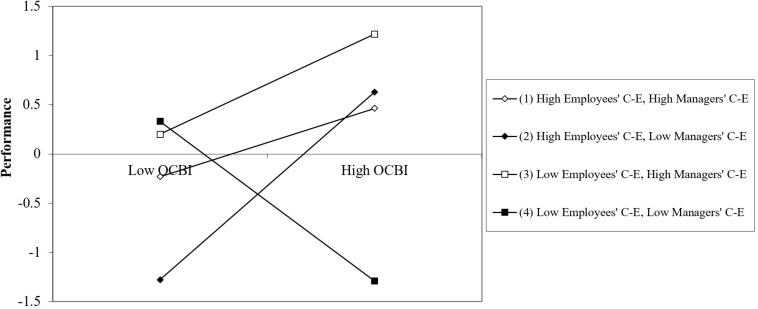
Impact of OCB on performance efficiency as a function of employees’ collective efficacy.

Figure 8 provides a graphic representation of the results of the three-way interaction.

As shown in Table 6 and Figure 8, the performance efficiency findings were similar to those reported for overall performance. OCB predicted performance for high values of employees’ CE, regardless of their managers’ CE and for moderate employees’ CE when their managers’ CE was either high or moderate, but not when their managers’ CE was low. Moreover, when both employees and managers had low CE, OBC had negative effects on performance, possibly due to frustration^4^.

**FIGURE 9:**
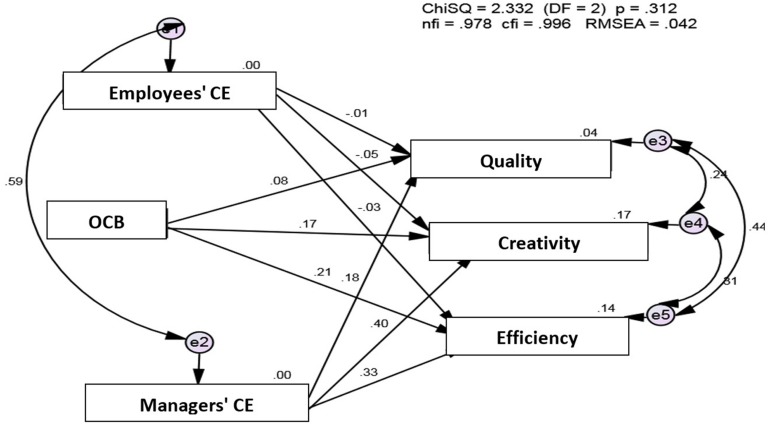
SEM of OCB as a predictor of three performance facets moderated by employees’ and managers’ collective efficacy.

Structural equation modeling (SEM) was used for a comprehensive examination of the model. As recommended, a measurement model was examined. It was made up of 6 constructs: OCB, CE (evaluated by managers and employees) and three performance dimensions (quantity, creativity, efficiency). A confirmatory factor analysis (CFA) was used. The ACFA model exhibited good fit with the data [χ^2^ = 2.33 (DF = 2); *p* = 0.312; RMSEA = 0.042; CFI = 0.996; NFI = 0.978], supporting the main hypotheses predicting performance dimensions (Figure 9).

As can be seen in Figure 9, the results of the SEM revealed that managers’ CE was the most important determinant of all the criteria of job performance. Thus, the role of managers’ beliefs in team resources appears to have significant and specific importance, leading to better performance.

## Discussion

The results of Study 2 supported the hypotheses that managers’ and employees’ CE would moderate the OCB-performance associations. This findings is consistent with the literature that has emphasized the important role of team efficacy on performance (Myers et al., 2004; Salanova et al., 2014). The findings revealed that managers’ CE had the greatest influence on employees’ performance. This finding is consistent with the literature that has underscored team managers’ prime role in facilitating performance (Pak and Kim, 2018).

## General Discussion

These two studies provide empirical evidence that supports a more accurate and comprehensive picture of the ways prosocial behaviors (OCB) facilitate or inhibit performance. Study 1 supported the first hypothesis that OE, which is related to employees’ beliefs in their internal resources, is a core antecedent of OCB. Study 2 supported the second hypothesis that the CE of both managers and employees, which represents employees’ beliefs in human external resources, moderates the OCB-performance associations. Using SEM, managers’ CE was shown to have the greatest influence on employees’ performance. By using a manager-employee dyadic model, the findings of a positive association between OCB and performance contributed to controlling for potential common method bias. The empirical support for the hypothesis regarding the moderating role of CE of both employees and managers sheds light on the ways in which employees’ OCB affects performance, and identifies the possible relative importance of employees’ and managers’ beliefs. The results also point to the conditions that are the most conducive to positive associations between prosocial behavior and performance, and the conditions that can lead to a negative association between OCB and performance (i.e., low CE among employees for qualitative performance). Finally, in addition to an examination of performance as a single construct, as has generally been the case in the vast majority of publications on this topic, we also examined the three dimensions of performance (quality, creativity and efficiency) that have been found to be crucial for achieving the best overall performance in today’s competitive markets (Miron et al., 2004). This separate analysis for each dimension leads to a better grasp of the specific effects of both employees’ and managers’ CE as moderators of OCB effects on performance.

Specifically, employees’ OCB positively predicted performance even after controlling for demographics. These findings are in line with previous works on the positive effects of OCB on performance (Choi, 2009; Lam et al., 2016; Park, 2018; Germeys et al., 2019). In addition, this positive association was only found when employees strongly believed in the ability of their work team (high employee CE) or when their managers strongly believed in their employees’ team abilities (high manager CE) but disappeared when employees, managers or both had weak beliefs in their teams (low CE). Under these conditions of low CE on the part of both employees and managers, OCB became an obstacle to achieving better performance quality. This effect may have been driven by frustration that can emerge when helping behaviors are met by low beliefs of employees and managers in their team’s abilities. Further empirical work should thus be conducted on this topic.

These results are also consistent with the important role of beliefs in team efficacy (CE) on performance as found in the literature (Myers et al., 2004; Salanova et al., 2014). The findings that managers’ CE had the most influence on employees’ performance is consistent with the literature that stresses team managers’ prime role in facilitating performance (Pak and Kim, 2018). In addition, when both employees’ and managers’ CE were high, OCB had the strongest positive effect on performance. Moreover, whereas performance was significantly higher for high OCB employees with high CE than for high OCB employees with low CE, the opposite was found when OCB was low. For low OCB employees, high CE led to lower performance, possibly due to frustration experienced when no assistance was provided by team members believed to have high abilities. Finally, whereas high OCB employees’ performance was significantly higher when managers had high CE than when managers had low CE, the opposite was found for low OCB employees. For low OCB employees, high managers’ CE did not lead to better employee performance than managers’ low CE.

The findings that OE serves as a core antecedent of OCB is consistent with previous findings on job self-efficacy and OCB in customer service employees (Reizer and Hetsroni, 2015) and extends these findings to employees’ overall performance and three performance dimensions. Thus, inducing employees to believe more fully in their inner job-related resources may facilitate their OCB. This might be examined by providing positive verbal feedback to employees on their successful performance at work.

The specific examination of the three performance dimensions revealed that the model was fully supported for performance efficiency and creativity, but less strongly for performance quality. Thus, prosocial behavior in organizations appears to have the greatest impact on performance efficacy and creativity. This finding supports our initial expectation that performance efficiency and performance creativity are more dependent on interactions with other employees whereas performance quality is more dependent on employees’ human capital and less dependent on interactions with other employees. The findings supported the main predictions and are consistent with studies that have examined CE from the employee point of view (Myers et al., 2004; Walumbwa et al., 2008; Lee and Ko, 2010; Salanova et al., 2014).

Overall, these data contribute to a better understanding of the mechanisms that facilitate the effects of prosocial behaviors on employees’ performance. In general, the stronger the beliefs in the abilities of employees’ teams team (high CE), the stronger the association between OCB and higher performance. The more strongly employees believe in their inner job-related resources, the more prosocial behavior they report.

### Research Implications

These studies make five theoretical contributions. They provide new insights into the power of OCB to predict performance using a manager-employee dyad when controlling for managers’ tenure in their position. Research findings, based mainly on employees’ self-reports, have shown that OCB has a predictive effect on performance (e.g., Choi, 2009), and that CE is predictor of performance as well (Myers et al., 2004; Salanova et al., 2014).

However, we found that although OCB has a predictive effect on performance, this effect disappears when the CE beliefs of both employees and their managers are low. Although many studies have examined OCB’s ability to predict performance, the literature review indicated that no study has tested the moderating effects of these associations. Here we used a relational perspective that captures the essence of these associations using the well-established CE concept.

Second, the studies here explored the role of OCB in performance from a relational perspective, which enriches current research frameworks. They examined prosocial behavior effects in work contexts more comprehensively by analyzing a broader spectrum of efficacy beliefs, which included the individual level (occupational-efficacy) and the group level of analysis (CE), which we believe is more relevant to the organizational context.

These studies extend the concept of CE to prosocial behaviors in the organization context. Although previous studies have found associations between group efficacy and collective action (Besta et al., 2017) and between CE and undergraduate students’ performance in teams (Brown, 2003), as far as we know, no study has explored or found associations between CE and prosocial behaviors in organizational contexts.

Fourth, using the well-established efficacy theory, OE was found to be an antecedent to OCB, thus providing a much more comprehensive theoretical grasp of the dynamics of psychological factors that contribute to better performance.

Finally, by not only examining one performance measure, but rather the core dimensions of performance, we were able to better pinpoint the specific areas of performance related to prosocial behavior. Using a dyad manager-employee model, we controlled for potential common method bias, since in Study 2, employees’ performance was evaluated by their managers rather than self-reported.

### Practical Implications

These data have implications for managers and organizations interested in achieving better performance. Improving CE beliefs is one way to do so. Tasa et al. (2007) noted that CE emerges in part through continual exchanges of information and observed behaviors within a team, and that CE is influenced by teamwork behaviors as a sum total. Brown (2003) found that verbal self-guidance training led to better CE and performance among undergraduate students. Thus, training both managers and employees in the use of verbal communication that reinforces their CE beliefs and strengthens employees’ beliefs in their inner job-related resources may enhance the associations between OCB and performance relations as well as between OE and OCB. In addition, developing teamwork and improving harmony in work teams may increase the likelihood that helping behaviors in the team will contribute to performance. Moreover, managerial feedback and evaluations conveyed to the team may affect their CE. Therefore, managerial training programs should place greater emphasis on the importance of feedback not only to individuals but also to teams as a whole. In addition, identifying managers’ CE, employees’ CE, and employees’ beliefs in their own job-related resources should improve placement planning that generates a stronger association between employees’ prosocial behavior and performance.

Moreover, if employees’ CE is low, encouraging OCB behaviors in this team may not lead to better performance. In these instances, the first step would be to facilitate employees’ beliefs in their team, which should strengthen the association between prosocial behavior in the team and its performance. This should also be empirically examined in future research using an experimental design. Finally, as prosocial behaviors, and both team-level and individual-level efficacy were found to be related to performance, additional steps should be taken to emphasize the organizational level, specifically by embedding and assimilating processes in the organizational culture that encourage helping behaviors and collective actions.

### Limitations and Suggestions for Future Research

Future research should be conducted using an experimental design to examine whether interventions to augment both CE and helping behaviors, as suggested here, also facilitate performance. In addition, helping behaviors in themselves, may increase the CE of team members. Thus, additional research should be conducted to explore the ways in which CE acts a mediator between prosocial behavior and performance. Moreover, although a well-established validated performance measure was used in both studies, and potential common method bias was controlled for, future research should use measures of actual rather than reported organizational performance as well.

## Conclusion

These two studies provide empirical support for a more comprehensive model that can better predict and understand the dynamics of prosocial behaviors within organizations and their effects on performance. Specifically, we found that to achieve the best performance from OCB, high CE on the part of both employees and managers are both crucial. In addition, OE emerged as one of the core antecedents of prosocial behaviors within organizations. These findings thus pave the way for theoretical and practical avenues of research that should be examined in future studies.

## Data Availability Statement

The datasets generated for this study are available on request to the corresponding author.

## Ethics Statement

Ethical approval for this research was obtained from the institutions where the study was conducted. Filling in the questionnaires was indicative of informed consent.

## Author Contributions

EY and JW made a substantial, direct and intellectual contribution to the work, and approved it for publication.

## Conflict of Interest

The authors declare that the research was conducted in the absence of any commercial or financial relationships that could be construed as a potential conflict of interest.

## Footnotes

1 It could be argued that OE moderates the OCB prediction of performance. Although not hypothesized, we empirically examined this possibility using the PROCESS macro (Hayes, 2013) model 1. Supporting our model (see Figure 1) no significant interaction was found between OCB X employees’ OE [*F*general performance (3, 111) = −1.83, *p* = 0.069; *F*quality(3, 111) = −1.68, *p* = 0.096; *F*creative(3, 111) = −1.67, *p* = 0.099]; *F*efficiency(3, 111) = 0.11, *p* = 0.914), This strengthens our model where OE serves as an antecedent to OCB and not as a moderator on the OCB prediction of performance.

2 Initially we also included gender in the analyses as an additional control variable when examining H3 and H4. Since similar results were found after controlling for gender, we present the analyses when controlling for managers’ tenure in the position alone.

3 It could be argued that OE, which was found in Study 1 to predict OCB, could also serve as a moderator for the effects of OCB on performance. Although not hypothesized, we empirically examined this possibility using the PROCESS macro (Hayes, 2013) model 3. Supporting our model (see Figure 2) no significant interaction was found between OCB X employees’ OE [*F*general performance(7, 79) = −0.53, *p* = 0.598; *F*quality(7, 79) = −0.09, *p* = 0.926; *F*creative (7, 79) = −1.33, *p* = 0.187]; *F*efficiency(7, 79) = 1.26, *p* = 0.212), OCB X managers’ OE [*F*general performance(7, 79) = 0.02, *p* = 0.983; *F*quality(7, 79) = 0.27, *p* = 0.786; *F*creative(7, 79) = 0.58, *p* = 0.561]; *F*efficiency(7, 79) = −1.57, *p* = 0.120), employees’ OE X managers’ OE [*F*general performance(7, 79) = −0.05, *p* = 0.960; *F*quality(7, 79) = −0.50, *p* = 0.616; *F*creative(7, 79) = 0.06, *p* = 0.954); *F*efficiency(7, 79) = −0.18, *p* = 0.855],or OCB X employees’ OE X managers’ OE [*F*general performance(7, 79) = −0.27, *p* = 0.788; *F*quality(7, 79) = −0.11, *p* = 0.916; *F*creative(7, 79) = −1.05, *p* = 0.300]; *F*efficiency(7, 79) = 1.37, *p* = 0.174) in predicting the general performance measure or the specific performance facets (quality, creativity, efficiency). This strengthens our claim that OE serves as an antecedent to OCB and not as a moderator on the OCB prediction of performance.

4 To examine whether similar results would be found after controlling for the effects of employees’ self-efficacy, we conducted all analyses as presented when also controlling for the employees’ self-efficacy measure. All results were similar to those reported, indicating that the moderation effects of collective efficacy were significant over and beyond the effects of employees’ beliefs in their personal general resources to achieve success.

## Appendix

OCB Items used in the two studies [from Williams and Anderson (1991)]

1. Assists others who have been away from the office or on leave.
2. Expresses willingness to take time to help others who have work-related problems.
3. Adjusts schedule to enable other employees to take time off.
4. Goes the extra mile to help newer employees feel welcome on the job.
5. Manifests real concern and courtesy to all, even under difficult business or personal situations.
6. Gives personal time to help others who have work or non-work issues.
7. Helps others do their work.
8. Lends own property to others to ease their workload.
